# Morphological Study of Fossa Ovalis in Formalin-Fixed Human Hearts and Its Clinical Importance

**DOI:** 10.3390/medicina57111254

**Published:** 2021-11-16

**Authors:** Monica Adriana Vaida, Caius Glad Streian, Cristina Gug, Nawwaf Sebastian Damen, Adelina Maria Jianu, Andreea Grigoriță, Laura Grigoriță

**Affiliations:** 1 Department of Anatomy and Embryology, “Victor Babeş” University of Medicine and Pharmacy, 300041 Timisoara, Romania; vaida.monica@umft.ro (M.A.V.); lauragrigorita@yahoo.com (L.G.); 2 Department of Cardiology, “Victor Babeş” University of Medicine and Pharmacy, 300041 Timisoara, Romania; streian.caius@umft.ro; 3 Department of Microscopic Morphology, “Victor Babeş” University of Medicine and Pharmacy, 300041 Timisoara, Romania; dr.cristina.gug@gmail.com; 4 Department of Pediatric Surgery, “Victor Babeş” University of Medicine and Pharmacy, 300041 Timisoara, Romania; damen.n.sebastian@gmail.com; 5 Faculty of Medicine, “Victor Babeş” University of Medicine and Pharmacy, 300041 Timisoara, Romania; andreea171994@yahoo.com

**Keywords:** anatomy, interatrial septum, fossa ovalis

## Abstract

*Background and Objectives:* Our study aimed to investigate the gross anatomy aspects of the fossa ovalis (FO) and the presence of some anatomical variation resulting from the incomplete fusion of septum primum and septum secundum, such as an atrial septal pouch (SP) and left atrial septal ridge. *Materials and Methods:* Thirty-one adult human hearts removed from formalin-fixed specimens were examined to provide information about the morphology of the FO. The organs were free of any gross anatomically visible pathological conditions. *Results:* The most common variants were the FO located in the inferior part of the interatrial septum (64.51%), circular (61.3%), with a net-like structure (51.62%), prominent limbus (93.55%), and patent foramen ovale (PFO) (25.8%). The right SP was observed in 9.67% of specimens, the left SP was observed in 29.03% of cases, and in 51.61% of cases, a double SP was observed. One sample presented a right SP and a double left SP, and one case showed a triple left SP, which was not reported previously to our knowledge. *Conclusions:* Knowledge of the interatrial septal anatomy becomes important for interventional cardiologists and should be documented before transeptal puncture.

## 1. Introduction

A true septum represents a partitioning wall shared by two structures [1], which can be removed without exiting the heart’s cavities [2]. Regarding the interatrial septum, in the literature, these are described as the atrial septum and the “true”, clinically significant, interatrial septum [3]. The classically atrial septum is bound superiorly by the ostium of the superior vena cava, inferiorly by the ostium of inferior vena cava, antero-inferiorly by the coronary sinus orifice, anteriorly by the right atrioventricular ring, and posteriorly by the folds of the atrial wall. The “true” atrial septum is represented by the valve of the foramen ovale and its anteroinferior base and represents 20% of the classically interatrial septum area [2]. During the third week of intrauterine life, the progenitor heart cells appear in the epiblast. They will become the primary heart area, a horseshoe-shaped cluster of cells located in the splanchnic mesoderm layer. These cells will form the paired cardiac tube. The primitive heart tube is formed by fusion of the two endocardial tubes on the 22nd day, which narrows and lengthens and is formed by five segments called the sinus venosus, primitive atrium, primitive ventricle, bulbus cordis, and truncus arteriosus. These primitive heart tubes will be partitioned by forming the atrial septum, atrioventricular septum, aorticopulmonary septum, and interventricular septum in four chambers [4]. Septation of the atrium begins with the formation of the septum primum, crescentic sagittal septum, which grows from the roof of the common atrium toward the atrioventricular septum, formed by the endocardial cushions developed in the atrioventricular canals [5]. The lower edge of the septum primum delimits with the endocardial cushions, an orifice, the ostium primum, which maintains the communication between the two atriums. The septum primum will fuse with endocardial cushions, but at the same time, another orifice, the ostium secundum, appears in the superior part of the septum primum. Initially, multiple secondary perforations occur in the septum primum, which will soon coalesce to form the single orifice, called the ostium secundum. The closure continues because another crescentic-shaped septum, the septum secundum, goes down from the superior surface of the right atrium, on the right side of the septum primum, and closes the ostium secundum partially. The opening left in the septum secundum is called the foramen ovale. It allows blood circulation from the right atrium to the left atrium during fetal life. The inferior part of the septum primum sends an expansion, the valve of foramen ovale, “the valve of Vieussens”, which is not attached and allows blood circulation during fetal life from the right atrium to the left atrium. After birth, the changes in the pulmonary circulation increase the left atrial pressure, which presses the valve of the foramen ovale against the septum secundum and closes the foramen ovale. The formation of multiple perforations in the septum primum is a characteristic of placental mammals, including humans [6]. In monotreme and marsupial mammals, birds, and reptiles, the perforations in the septum primum do not coalesce to form the ostium secundum. They are sealed by myocardial and endocardial growth after birth [7]. According to Anderson [2], the septum primum persists as the valve of the foramen ovale, the classical septum secundum represents an infolding from the atrial roof, which forms the superior part of the limbus of the fossa ovalis (FO), and the rest of the limbus is produced by the muscularization of the vestibular spine.

Our study supplements the knowledge of the morphology of the “true” interatrial septum, which is important in order to avoid exiting the heart during interventional cardiac procedures [8].

## 2. Materials and Methods

The study was conducted in the Department of Anatomy and Embryology, “Victor Babeş” University of Medicine and Pharmacy, Timisoara, and included 31 human hearts removed from formalin-fixed specimens. The hearts were free of any gross anatomically visible pathological conditions. The right atrium was opened by a classical incision [9], extending between the orifices of the superior and inferior vena cava and medially from the sulcus terminalis. The left atrium was opened by an H-shaped incision, composed of two vertical incisions between the left pulmonary veins and the right pulmonary veins, and one horizontal incision connecting the mid-point of the other two. After opening the right atrium, the FO was examined regarding its morphology and permeability, with the heart in the anatomical position. In addition, the following aspects of FO were noted and photographed:

- Position of the FO in the interatrial septum;
- Shape of the FO;
- Aspect of the FO;
- Presence and location of the limbus of the FO;
- Presence of the septal pouch (SP), right SP opened into the right atrium, left SP opened into the left atrium, and the position of the SP in interatrial septum;
- Presence of patent foramen ovale (PFO).

## 3. Results

Thirty-one formalin-fixed human hearts were carefully examined, with observations below on the morphology of the FO:

Regarding the position of FO in the interatrial septum, FO was positioned as inferior in 20 specimens (64.51%), anterosuperior in 2 specimens (6.45%), and in the middle of the interatrial septum in 9 specimens (29.03%).

**Figure 1:**
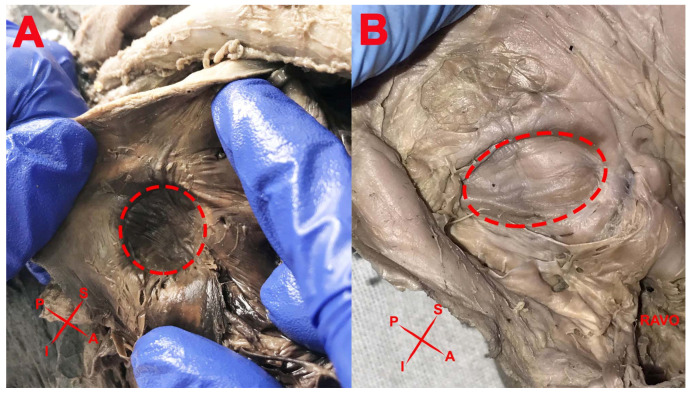
Shape of the FO: (**A**) Circular; (**B**) Oval. RAVO—right atrioventricular orifice.

The shape of the FO (Figure 1) was variable; namely, it was elliptical in 2 specimens (6.45%), oval in 10 specimens (32.25%), and circular in 19 specimens (61.3%).

The aspect of the FO was smooth in 15 specimens (48.38%) and has a net-like structure or was cribriform in 16 specimens (51.62%). Cribriform FO presents the fibrous-strands, different from the Chiari network, described in the literature as a mobile reticulated network structure originating from the Eustachian valve, connecting to other parts of the right atrium [10,11]. In the 16 specimens, these net-like structures were observed in:

(1) Six specimens (37.5%), on the entire surface of the FO;
(2) Six specimens (37.5%), on the posteroinferior part of the FO;
(3) Four specimens (25%), only on the inferior part of the FO.

**Figure 2:**
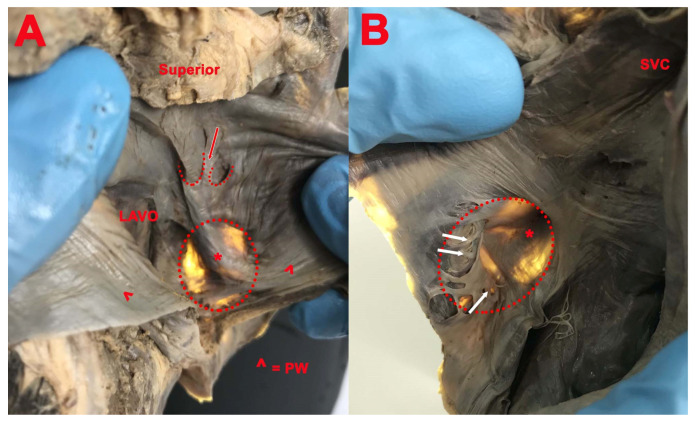
(**A**): Double SP with superior concavity; common fibro-muscular bundle (red arrow); *—right and left image of the prominent ridge along FO; *LAVO*—left atrioventricular orifice; *PW*—posterior wall. (**B**): SP in the superior part of the limbus; net-like structure in the opposite part of SP (white arrows); *SVC*—superior vena cava.

In one case (Figure 2), a prominent ridge along the FO was found.

Concerning the presence and location of the limbus or annulus of the FO, the limbus (annulus) was flat in 2 cases (6.45%) and prominent in 29 cases (93.55%).

The segment of the limbus or annulus that was prominent was:

(1) All around the circumference in 2 cases (6.89%);
(2) Superior in one case (3.44%);
(3) Superior and anterior in 3 cases (10.35%);
(4) Superior, posterior, and inferior in 13 cases (44.82%);
(5) Anterior, superior, and posterior in 7 cases (24.14%);
(6) Superior and posterior in 3 cases (10.35%).

**Figure 3:**
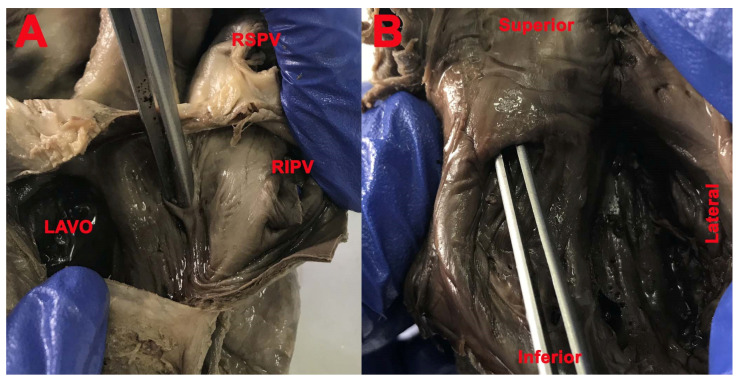
(**A**,**B**) SP (lifted by clip). (**A**): *LAVO*—left atrioventricular orifice; *RSPV*—right superior pulmonary vein; *RIPV*—right inferior pulmonary vein.

**Figure 4:**
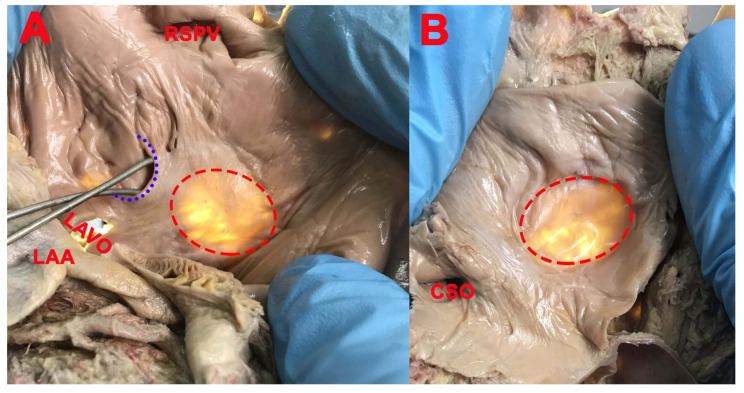
(**A**): SP (blue interrupted line); *LAA*—left atrial appendage (left auricle); *LAVO*—left atrioventricular orifice; *RSPV*—right superior pulmonary vein; (**B**): SP absent; FO (red interrupted line); *CSO*—coronary sinus opening.

The SP was bilateral (right and left double SP—Figure 3) in 16 cases (51.61%), in 3 cases only on the right side (9.67%), in 9 cases only on the left side (Figure 4) (29.03%), and in 3 cases absent (9.68%).

(1) Right and double SP had the opening in the right atrium in 19 cases (61.29%) and the concavity was located in the inferior part (12 cases, 63.15%), anteroinferior part (4 cases, 21.05%), and posteroinferior part (3 cases, 15.8%) of the circumference of the FO;
(2) Left and double SP had the opening in the left atrium in 25 cases (80.64%). The concavity was located in the superior part (15 cases, 60%), posteroinferior part (2 cases, 8%), posterosuperior part (2 cases, 8%), anterior part (3 cases, 12%), posterior part (1 case, 4%), and anterosuperior part (2 cases, 8%) of the circumference of the FO.

**Figure 5:**
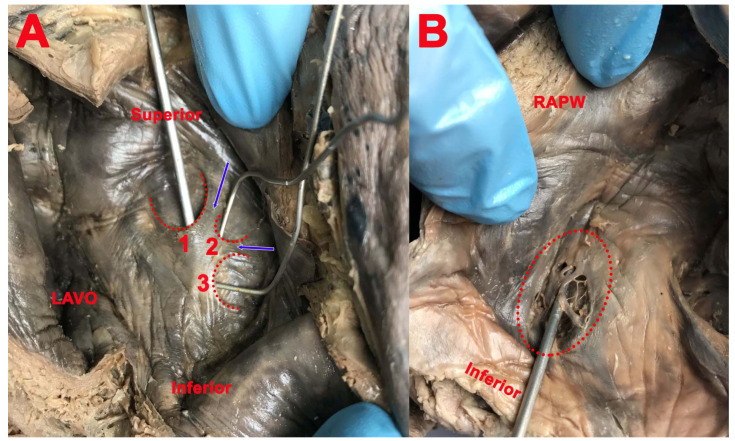
(**A**): 1, 2, and 3—triple SP, separated by two fibro-muscular bundles (blue arrows); *LAVO*—left atrioventricular orifice. (**B**): FO (red interrupted line) covered by net-like structure; *RAPW*—right atrial posterior wall.

A triple left SP (Figure 5) was found in one case, separated by a fibro-muscular bundle with four horns. Two of the left SPs were superficial with the concavity posteroinferior and posterior, and one was superficial with the concavity superior. All three cavities had the apex orientated downward, at the same level, and there was no communication between them.

In one case (Figure 2), we found a right SP in the superior part of the limbus, with inferior concavity and the apex orientated upward, as well as a double left SP, both with superior concavity and the apex orientated downward, delimitated by three fibro-muscular bundles, one common, one anterior, and one posterior. 

**Figure 6:**
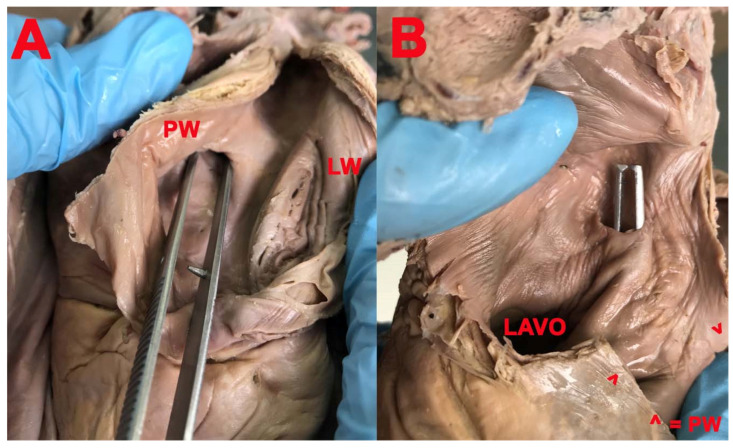
(**A**,**B**): PFO. (**A**): *PW*—posterior wall; *LW*—lateral wall. (**B**): *LAVO*—left atrioventricular orifice; *PW*—posterior wall.

PFO (Figure 6) was present in six cases (19.35%), in which the aspect of FO was smooth. The SP had the opening in the right atrium and was located in the superior part of the circumference of the FO, as well as in two cases in which the aspect of FO was the net-like structure (6.45%).

## 4. Discussion

Regarding the cribriform aspect of the FO, we found an unusual characteristic in six cases in which the entire surface of the FO had a net-like structure. The net-like appearance of the posterior and inferior parts of the FO represents multiple fenestrations in the septum primum. The net-like appearance on the entire surface of the FO represents fenestration of both the septum primum and the septum secundum. The cribriform aspect of the FO can be the consequence of incomplete overlap or atrophy of the septum primum or the septum secundum [3], considering the formation of ostium secundum is preceded by the appearance of multiple perforations in the septum primum. These cribriform aspects of the FO could represent an obstacle in transeptal puncture, may cause difficulties in transcatheteric closure of PFO [12], and according to Klimek-Piotrowska et al. [3], could be seen as a bilaminated interatrial septum, with a twofold line of the interatrial septum wall with a contrast layer inside. In a foregoing publication, cases were shown to have partially fenestrated FOs but with a central defect in the center of FO [13], an aspect that we did not find.

Regarding the presence of a prominent ridge along the FO, this was described with a prevalence of 18.7% in pig hearts, 15% in ovine hearts [14], and in humans by Shizukuda et al. [15] in one case and by Zisa et al. [16] in five human hearts on three-dimensional transesophageal echocardiography and may represent a challenge in the transseptal puncture. Zisa et al. [16] discussed that these anomalous ridges of the left side of the interatrial septum may depend on an irregular fusion between the septum primum and the septum secundum and is different from a prominent rim of a PFO.

The limbus of FO, also called the ‘annulus ovalis’ (Eustachii, 1564 quoted in Paraskevas et al.) [17] or the ‘oval ring of Vieussens’ (Vieussens, 1705, mentioned in Paraskevas) [17], is described as the crescentic upper margin of the FO, prominent above and in front of the fossa. It indicates the lower edge of the septum secundum of embryonic hearts [18].

The atrial SP has been a much-discussed concept from the moment of its discovery. According to Krishnan and Salazar [19], the SP represents a pocket accessible from the left atrium or the right atrium or located on both sides of the interatrial septum [20] in the absence of a PFO, as a result of incomplete fusion of the septum primum and septum secundum. The left SP is composed of an atrial wall, a free wall represented by the remnant valve of the FO or falx septi with a falciform aspect with concavity directed anteriorly and upwards [18], an orifice, a lumen, and an apex [20]. The right SP is smaller than the left SP [20] and presents an apex, an orifice, a lumen, and it is bounded in its anterosuperior part by the limbus of the FO. The right SP is the consequence of incomplete fusion between the septum primum and septum secundum, fusion limited to the cranial part of the overlap area, the left SP is the consequence of fusion limited in the caudal part of the overlap area, and the double SP is the consequence of fusion limited to the central part of the overlap area, [19,21]. According to Holda [21], the prevalence of SPs increased among older subjects. The PFO increased among the younger subjects because, during life, the PFO evolves into a SP or a smooth septum. A triple left SP in autopsied material had not been reported previously. Regarding the musculo-fibrous bundles that surround the cavity of the left SP, as were found in the present case, Holda et al. [21] discussed that one of the protective mechanisms against SP thrombosis is represented by the contraction of these fibers, which can promote emptying of the pouch. On the other hand, a factor that may favor the left SP’s stasis is represented by muscle contraction in the SP orifice that prevents the SP from emptying and the communication between the left atrium and the SP cavity [21]. The right SP seems to be just an anatomical variation with no clinical significance [20]. The left SP may be responsible for blood stasis and thrombus formation [21]. In 25% to 30% of patients with an ischemic stroke, the cause or source of the stroke may not be clear. Hence these cases are commonly termed cryptogenic strokes. These represent a definite knowledge gap in the field, motivating physicians to examine the prevalence of the pouch in patients who have already had strokes, especially those with obscure etiology. It is believed that, like the left atrial appendage, atrial septal aneurism, and PFO, the SP represents an additional structure that can cause thromboembolic strokes [22].

Case reports and epidemiologic results have demonstrated thrombus within the left SP and the relation to cryptogenic stroke. Initial results were mixed, with one study showing, and others not showing. an association between the left SP and cryptogenic stroke, requiring further evaluation, particularly in the younger patients suffering from a stroke. Although patients with interatrial abnormalities and stroke have lower thresholds for the induction of atrial fibrillation, this arrhythmia is rarely documented in patients with cryptogenic stroke and a PFO [23,24].

Later studies have demonstrated an association between the presence of the left SP and cryptogenic stroke. The left SP could thus be considered a possible site of thrombus formation. A further meta-analysis demonstrated the association between the left SP and cryptogenic stroke. The univariate analysis showed that the risk of cryptogenic stroke is higher among patients with a left SP than in cases without a left SP [25,26,27,28].

The pathogenesis of cryptogenic stroke in patients with interatrial septal abnormalities is not entirely understood. The morphologic characteristics of the interatrial septum that best predict the risk of thromboembolism remain to be further clarified.

The present recommendation is that any left SP finding should be noted during transesophageal echocardiography and thoroughly investigated, ideally with multiplane/tridimensional imaging to exclude thrombus. No clear consensus exists about whether the presence of a left SP alone in cryptogenic stroke is an indication for anticoagulation or other treatment [29,30].

Regarding the co-existence between the left SP and PFO, considering that both the left SP and PFO represent developmental defects in the interatrial septum (a left SP being the remnant of an incompletely fused septum primum and septum secundum and PFO is the sequel of an unfused septum primum and septum secundum), the study of Terpenning et al. on 275 cardiac CT found the prevalence of PFO in patients with a left SP to be 5.9% [31].

## 5. Conclusions

Knowledge of the morphology of the FO becomes essential during cardiac surgery, especially the net-like structure of FO and the presence of the SP, which can create complications during the transseptal puncture and catheter manipulation.

## Data Availability

No new data were created or analyzed in this study. Data sharing is not applicable to this article.

## References

[1] Asirvatham S.J., Stevenson W.G. (2013). Editor’s perspective: The interatrial septum. Circ. Arrhythm. Electrophysiol..

[2] Anderson R.H., Brown N.A., Webb S. (2002). Development and structure of the atrial septum. Heart.

[3] Klimek-Piotrowska W., Hołda M.K., Koziej M., Piątek K., Hołda J. (2016). Anatomy of the true interatrial septum for transseptal access to the left atrium. Ann. Anat.

[4] Dudek R.W. (1996). High.-Yield Embryology.

[5] Sadler T.W. (2015). Cardiovascular system. Langman’s Medical Embryology.

[6] Jensen B., Wang T., Moorman A.F.M. (2019). Evolution and Development of the Atrial Septum. Anat. Rec..

[7] Jensen B., Spicer D.E., Sheppard M.N., Anderson R.H. (2017). Development of the atrial septum in relation to postnatal anatomy and interatrial communications. Heart.

[8] Naqvi N., McCarthy K.P., Ho S.Y. (2018). Anatomy of the atrial septum and interatrial communications. J. Thorac. Dis..

[9] Papilian V., Papilian V.V. (1994). Manual Practic de Disecție și Descoperiri Anatomice.

[10] Kerut E.K., Norfleet W.T., Plotnick G.D., Giles T.D. (2001). Patent foramen ovale: A review of associated conditions and the impact of physiological size. J. Am. Coll. Cardiol..

[11] Loukas M., Sullivan A., Tubbs R.S., Weinhaus A.J., Derderian T., Hanna M. (2010). Chiari’s network: Review of the literature. Surg. Radiol. Anat..

[12] Joshi S.D., Chawre H.K., Joshi S.S. (2016). Morphological study of fossa ovalis and its clinical relevance. Indian Heart J..

[13] Chan K.C., Godman M.J. (1993). Morphological variations of fossa ovalis atrial septal defects (secundum): Feasibility for transcutaneous closure with the clam-shell device. Br. Heart J..

[14] Hołda M.K., Pietsch-Fulbiszewska A., Trybus M., Koziej M. (2018). Morphological variations of the interatrial septum in ovine heart. PLoS ONE.

[15] Shizukuda Y., Muth J., Chaney C., Attari M. (2012). Anomalous ridge on the left atrial side of the atrial septum. Ann. Card Anaesth..

[16] Zisa D., Faletra F.F., Wessler B.S., Halin N.J., Reddy P., Patel A.R., Pandian N.G. (2019). Ridges and Pouches: A Case Series of Anomalous Atrial Septal Fusion. Case.

[17] Paraskevas G., Koutsouflianiotis K., Iliou K. (2017). The first descriptions of various anatomical structures and embryological remnants of the heart: A systematic overview. Int. J. Cardiol..

[18] Filipoiu F.M. (2014). Atlas of Heart Anatomy.

[19] Krishnan S.C., Salazar M. (2010). Septal pouch in the left atrium: A new anatomical entity with potential for embolic complications. JACC Cardiovasc. Interv..

[20] Mazur M., Jasinska K.A., Walocha J.A. (2018). The morphology, clinical significance and imaging methods of the atrialseptal pouch: A critical review. Transl. Res. Anat..

[21] Hołda M.K., Koziej M., Hołda J., Piątek K., Tyrak K., Chołopiak W., Bolechala F., Walocha J.A., Klimek-Piotrowska W. (2016). Atrial septal pouch—Morphological features and clinical considerations. Int. J. Cardiol..

[22] Hughes S. (2010). The Atrial Septal Pouch—A New Source of Thrombus?. Medscape.

[23] Berthet K., Lavergne T., Cohen A., Guize L., Bousser M.G., Le Heuzey J.Y., Amarenco P. (2000). Significant association of atrial vulnerability with atrial septal abnormalities in young patients with ischemic stroke of unknown cause. Stroke.

[24] Tugcu A., Okajima K., Jin Z., Rundek T., Homma S., Sacco R.L., Elkind M.S.V., Di Tullio M.R. (2010). Septal pouch in the left atrium and risk of ischemic stroke. JACC Cardiovasc. Imaging.

[25] Hołda M.K., Koziej M. (2018). Left-Sided Atrial Septal Pouch as a Risk Factor of Cryptogenic Stroke: A Systematic Review and Meta-Analysis. Cereb. Dis..

[26] Kuwaki H., Takeuchi M., Kaku K., Haruki N., Yoshitani H., Tamura M., Okazaki M., Abe H., Otsuji Y. (2011). Thrombus attached to the left atrial septal pouch assessed on 3-dimensional transesophageal echocardiography. Circ. J..

[27] Loza G., Américo C., Gómez A., Janssen B., Pazos A., Parma G., Florio L. (2019). Prevalence of septal pouch in a cohort derived for transesophageal echocardiography. Rev. Urug. Cardiol..

[28] Wong J.M., Fisher M. (2016). The potential role of the left atrial septal pouch in cryptogenic stroke. Expert Rev. Cardiovasc..

[29] Kabirdas D., Nekkanti R. (2018). Webbed left atrial septal pouch-A new anatomical variant. Echocardiography.

[30] Strachinaru M., Castro-Rodriguez J., Verbeet T., Gazagnes M.D. (2017). The left atrial septal pouch as a risk factor for stroke: A systematic review. Arch. Cardiovasc. Dis..

[31] Terpenning S., Ketai L.H., Rissing S.M., Teague S.D. (2015). Correlation of Left Atrial Septal Pouch with the Prevalence of Patent Foramen Ovale: A Retrospective Review. Cardiol. Angiol. Int. J..

